# Knowledge of the community regarding mental health problems: a cross-sectional study

**DOI:** 10.1186/s40359-021-00607-5

**Published:** 2021-07-14

**Authors:** Yonas Tesfaye, Liyew Agenagnew, Susan Anand, Gudina Terefe Tucho, Zewdie Birhanu, Gutema Ahmed, Masrie Getnet, Kiddus Yitbarek

**Affiliations:** 1 Department of Psychiatry, Jimma University, Po Box 378, Jimma, Oromia Ethiopia; 2 School of Nursing and Midwifery, Jimma University, Jimma, Oromia Ethiopia; 3 Department of Environmental Health Sciences and Technology, Jimma University, Jimma, Oromia Ethiopia; 4 Department of Health, Behavior, and Society, Jimma University, Jimma, Oromia Ethiopia; 5 Department of Biostatistics and Epidemiology, Jimma University, Jimma, Oromia Ethiopia; 6 Department of Health Policy and Management, Jimma University, Jimma, Oromia Ethiopia

**Keywords:** Knowledge, Perception, Mental health problems, Community, Jimma

## Abstract

**Background:**

Knowledge of the community regarding mental health problems has a remarkable impact on the attitude, the help-seeking path, and prevention of stigma and discrimination against patients with mental health problems. It is also the cornerstone for designing evidence-based community mental health interventions. However, the evidence is scarce in developing countries like Ethiopia. This study aimed to assess the knowledge regarding mental health problems and associated factors among communities of Jimma Zone, Oromia, Ethiopia.

**Methods:**

A community-based cross-sectional study was conducted in the Jimma zone from March 1 to 22, 2020. A structured, pretested, and interviewer-administered questionnaire was used to collect data from 420 study participants selected through a systematic sampling technique. The knowledge about mental health was measured by the adapted version of the Mental Health Knowledge Schedule tool. Data were entered into Epi-data version 3.1 and exported to SPSS version 23.0 for analysis. Multivariate logistic regression analysis was done, and *p*-value < 0.05 and 95% CI were used to determine the predictors of the outcome variable.

**Results:**

The overall knowledge score showed (188, 44.8%) of the respondents had inadequate knowledge. Moreover, (75, 17.9%) of the respondents reported psychiatric disorders are contagious, and (138, 32.9%) mentioned leaving alone is the treatment for mental illness. Talking or laughing alone and showing strange or unusual behaviors were described as symptoms of mental illness by the majority (407, 96.9%) and (403, 96.0%) of respondents, respectively. Brain dysfunction was attributed to the cause of mental illness by most (390, 92.9%) of the study participants. Similarly, the percentage of responses that attributed the causes of mental illness to possession by an evil spirit, God’s punishment, and witchcraft were significantly high (368, 87.6%), (321, 76.4%) and (259, 67.1%), respectively. Furthermore, regression analysis showed that respondents who were able to read and write were 64% less likely to have adequate knowledge than those in secondary school and above educational status (AOR = 0.34, 95% CI (0.16–0.69)).

**Conclusion:**

Knowledge of mental illness among the general public was relatively poor and higher levels of education were associated with good knowledge of mental health problems; this suggests the need for due emphasis on public education to improve the mental health literacy status of the community.

**Supplementary Information:**

The online version contains supplementary material available at 10.1186/s40359-021-00607-5.

## Background

Mental health and physical and social health are closely interwoven fibers that are vital for every individual. Mental health extends beyond diagnosed mental disorders. According to World Health Organization (WHO) (2002), it encompasses personal well-being, perceived self-efficacy, autonomy, competence, intergenerational dependence, and recognition of the ability to realize one's intellectual and emotional potential [1–4].

Mental health is essential for the overall well-being of individuals, societies, and countries. Despite being imperative for human existence, in many countries, unlike the importance given to physical health, mental health has been relegated. This could partly be due to the myths and misconceptions surrounding mental illnesses in many parts of the world [5–7].

Negative views, fear, and stigmatization are responsible for the ostracization of mentally ill persons and lack of access to proper psychiatric treatment Therefore, mentally ill people seek professional help from hospitals after they have tried all options and the symptom has worsened. This negatively affects the prognoses of treatment [8, 9]. Mental disorders are attached to negative and stigmatizing attitudes, often due to a lack of knowledge about mental illness [10, 11]. People with mental illness are perceived as dangerous, unpredictable, and different from others, responsible for their condition, hard to talk to, and incurable in the community [12–14].

The concept of mental health problems and its causes vary in different communities [5, 15]. Society's knowledge regarding mental illness is often far from the scientific view, and it negatively affects treatment-seeking and adherence [9].

People's perception about mental ailments is influenced by their knowledge, encounters with people suffering from mental illnesses, media portrayal, cultural stereotypes, and their personal experiences of mental disorders [16, 17]. In many African societies, psychiatric illness is believed to be either an outcome of a familial defect or the 'handiwork of evil machinations' (demons, evil spirits). It is not uncommon to blame psychiatric patients for their illness, especially when it is an alcohol and/or substance-related problem [18, 19].

Literature shows Ethiopians, like other sub-Saharan African societies, attribute mental illnesses almost exclusively to supernatural phenomena. Mental illnesses have predominantly been attributed to possession by evil spirits, punishment from God or guardian spirits for sins, and/or curses, spells, or bewitchment by people alleged to have supernatural powers. Research also points out the limited and poorly developed infrastructure for modern mental health services [20–22].

Help-seeking behavior is influenced by people's knowledge of mental health problems and illness, which are based on the combinations of traditional and modern beliefs. Mental health knowledge includes the capacity to identify mental health problems, understanding of risk factors and causes, professional help available, attitudes that promote recognition, and appropriate mental health help-seeking behaviors [23–26].

Often mental illnesses are poorly understood by the general public, which threatens the effectiveness of patient care and rehabilitation [27, 28]. Beliefs about the causation and experience may influence patients' beliefs about effective treatment and may also determine the type of treatment that is sought. At times, the mentally ill are blamed for bringing on their illness. In contrast, others may see mentally ill people as victims of unfortunate fate, religious and moral transgression, or even witchcraft [29]. These misbeliefs may lead to denial of the problem by both the sufferers and their families, with subsequent delays in seeking professional treatment [28, 30–32].

Scientific evidence demonstrates that low rates of seeking psychiatric help are mainly due to inadequate knowledge of mental health disorders, including mental disorders symptoms, and psychiatric treatments [33]. Evidence revealed improved knowledge about mental health and illness leads to less stigma [34–36].

Heterogenous causation of biological, psychological, social, and cultural causes of mental illness was reported from the studies done in Nigeria [37–39], northern Ethiopia [13], and Denmark [40]. Whereas biological factors were reported as the main causes of mental illness in the studies conducted in Tanzania [41], Eretria [38], and Saudi Arabia [42]. Conversely, in South Africa [43], elsewhere in Ethiopia [44–46], Nigeria [36], and, Pakistan [47] mental illness was attributed to religious and cultural factors,

While a combination of medical, religious, and traditional treatment was favored by the respondents in the studies done in Eritrea [48], Saudi Arabia [49], Nigeria [39], and Ethiopia [5], medical management (medication and counseling) was the most preferred among the study subjects in Tanzania [41], Saudi Arabia [42], Slovak Republic, and Russia [50], New Zealand [51], South Africa [43], Ethiopia [13, 52], and India [45].

Overall, inadequate mental health knowledge of the community was described in various studies conducted in many parts of the world [9, 28, 30, 37, 38, 40, 41, 53–55]. The differences in the explanation of the causation and management of mental illness could be due to the difference in the literacy status, socio-cultural-economic disparities, availability, and use of media, and the difference in the availability of mental health services among the countries.

To the best of our knowledge, only a few studies were conducted regarding the rural communities' understanding of mental health problems in Ethiopia. The previous studies were done a long time ago, with fewer sample size, and conducted in different geographical-socio-cultural contexts [9, 44, 46, 56]. Hence, assessing the community's knowledge is essential in designing an appropriate mental health promotion and scaling up the public's utilization of mental health services. Therefore, the present study aimed to assess the knowledge and associated factors of the Jimma zone community towards mental health and mental health problems.

## Methods and materials

### Study setting and period

The study was conducted in Jimma zone, Seka chekorsa district. Jimma zone is divided into 20 districts and one town administration. The total population of the zone was 3,209,127 in 2017 [57]. There are four primary hospitals, 20 health centers, and 117 health posts [39] in the zone. A total of 1024 health extension workers are serving the population of this area. Seka chekorsa district is located 20 km from Jimma town, and the district has 30 kebeles (lowest administrative division) with a total population of 208,096 [58]. This district is resourced with a primary hospital, nine health centers, and 35 health posts. The study was conducted from March 1 to 22, 2020.

### Sample size

The sample size was calculated by using a single population proportion formula. We have taken the estimated proportion of community knowledge regarding mental health problems 50%, 95% confidence level, 5% margin of error. Accordingly, n = (zα/2)^2^ P(1 − p)/d^2^. Calculating the sample size n = (1.96)^2^ × 0.5 (1–0.5)/(0.05)^2^ become 384. Finally, with the addition of a 10% contingency for non-response, the final sample size was 423 households.

### Sampling procedures

A community-based quantitative cross-sectional survey was conducted. First, the Seka chekorsa district was selected from 20 districts in the zone through a lottery technique. Out of the 30 kebeles in this district, nine were determined by lottery method based on the World Health Organization (WHO) sample size calculation guideline in the district health system [59]. The proportional allocation determined the number of sampled respondents from each kebele to the total number of households in each selected kebeles. Systematic random sampling technique was used to select the study units, and periodic interval (K) was calculated using the formula K = N/n, whereby N is the total households in the selected kebeles (1555), and n is the estimated sample size (423). Accordingly, every four households were included in the study. The first study unit was selected by a lottery method between the 1st and 4th households. Finally, randomly selected household members aged 18 and above and living in the district for six months or more were interviewed.

### Inclusion and exclusion criteria

All the study community members age 18 and above and living in the district for six months or more were included in the study. Community members with acute or chronic illnesses which would interfere with providing accurate information were excluded.

### Measurements and procedures

The questionnaire consisted of sociodemographic and mental health knowledge questions. The knowledge of the community regarding mental health problems was measured by the adapted version of the Mental Health Knowledge Schedule (MAKS) with a “Yes” or “No” response (Additional file 1). The survey tool has good reliability and validity across many works of literature [60–62]. The reliability, Cronbach alpha score of the scales in this study was 0.66. The median score was calculated for knowledge-related questions. A cutoff point below and above the median score was considered to estimate the proportion of the community with adequate and inadequate knowledge. Twenty-three health extension workers collected data after getting training for two days on the questionnaire's content, data recording, and the ethical principles of data collection. The data were obtained through a face-to-face interviewer-administered questionnaire prepared in English and translated into Afaan Oromo, and back-translated to English to ensure its consistency by blinded language experts. The translation was face validated by two independent external experts in the field. Moreover, the questionnaire was pre-tested on 5% of the population in another district to check the impending problems of the data collection tool.

### Statistical analysis

The collected data were cleaned, coded, entered into Epi Data version 3.1, and exported to SPSS version 23 for analysis. Descriptive statistics were done to summarize the dependent and independent variables. The logistic regression analysis model was used to identify the factors associated with the outcome variable; first Bivariate logistic regression was done, and variables with a *p*-value < 0.25 were selected as candidate variables for multivariate logistic regression analysis. Multicollinearity and Lemeshow-Hosmer test of model fitness test was done before the final model. Then, variables with *P*-value < 0.05 and 95% confidence interval odds ratio were considered factors associated with the outcome variable.

## Results

### Socio-demographic characteristics

A total of 420 study participants were interviewed successfully, giving a response rate of 99.3%. The mean age of the respondents was 37.2 (SD ± 11.9) with a range of 18 to 80 years. Most of the study participants were females (230, 54.8%), married (345, 82.1%), from rural residence (253, 60.2%). Additionally, most of the study participants were from the Oromo ethnic group (395, 94%) and Muslims (384, 91.4%). The mean family monthly income was 1562.5 Ethiopian Birr (ETB) (SD ± 2769.8) (Approximately $48.00 US), and most respondents were unable to read and write (168,40%) (Table 1).

**Table 1:** Sociodemographic characteristics of respondents at Jimma Zone, Seka woreda South-west Ethiopia, March 2020

Variables	Characteristics	Frequency	Percentage
Sex	Male	190	45.2
	Female	230	54.8
Age	37.2 (SD ± 11.9) Range 18 to 80 years		
Residence	Urban	167	39.8
	Rural	253	60.2
Birth order	First	183	43.6
	Second	102	24.3
	Third	60	14.3
	4 or more	75	17.9
Ethnicity	Oromo	395	94.0
	Amhara	12	2.9
	Others*	13	3.1
Religion	Muslim	384	91.4
	Orthodox	24	5.7
	Others*	12	2.8
Family monthly Income	1562.5 (SD ± 2769.8)		
Educational status	Unable to read and write	168	40.0
	Read and write	81	19.3
	Primary school (1–8)	112	26.7
	Secondary school (9–12)	49	11.7
	Diploma	6	1.4
	Degree and above	4	1.0
Marital status	Single	30	7.1
	Married	345	82.1
	Divorced	12	2.9
	Widowed	33	7.9
Occupational status	Farmer	219	52.1
	Merchant	61	14.5
	Daily laborer	18	4.3
	Housewife	100	23.8
	Private employed	2	0.5
	Government employed	20	4.8

*Other religion includes Wakefata and Catholic

*Other Ethnicity includes Kaffa, Yem, Guraghe

### General knowledge of the respondents regarding mental health problems

Almost all (404, 96.2%) of the study participants knew psychiatric disorders are a kind of medical disorder, and the majority (362, 86.2%) recognize psychiatric disorders as treatable. On the contrary, (75, 17.9%) of the study participants reported psychiatric disorders are contagious, and (138, 32.9%) described leaving alone as a treatment for mental illness. Additionally, (155, 36.9%) of the respondents were rejected that mental health problems are identical for males and females. Talking or laughing alone and showing strange or unusual behaviors were reported as the major symptoms of mental illness by (407, 96.9%) and (403, 96.0%) of the study participants, respectively (Table 2).

**Table 2:** Knowledge of the respondents towards mental health and mental health problems at Jimma Zone, Seka woreda South-west Ethiopia, March 2020

Variables	Characteristics	Response	Frequency	Percentage
Psychiatric disorders are a kind of medical disorders		Yes	404	96.2
Psychiatric disorders are contagious diseases		Yes	75	17.9
Recovered psychiatric patients are employed productively		Yes	303	72.1
Psychiatric disorders are treatable		Yes	362	86.2
People with severe mental health problems can fully recover		Yes	88	20.9
Leaving alone is the treatment for mental illness		Yes	138	32.9
Which are symptoms of mental illness	Irritability	Yes	352	83.8
	Talking/laughing alone	Yes	407	96.9
	Wandering	Yes	393	93.6
	Excessive and unusual happiness	Yes	339	80.7
	Strange/unusual behavior	Yes	403	96.0
	Excessive Feeling sad, tearful	Yes	376	89.5
	Aggression/violence	Yes	401	95.5
	Hearing and seeing things that are not there	Yes	389	92.6
	Lack of sleep	Yes	376	89.5
	Talkativeness	Yes	394	93.8
	Trying to kill oneself	Yes	386	91.9
	Isolating oneself	Yes	401	95.5
Older people may develop mental disorders		Yes	371	88.3
Children may develop mental disorders		Yes	332	79.0
Women may develop mental disorders as equal to male		Yes	265	63.1
Lower socioeconomic class increases the risk of having psychiatric disorders		Yes	310	73.8

Responses only “Yes” or “No”

### Knowledge of the respondents regarding the cause and treatments of mental health problems

Among the causes of mental illness, the most significant responses were brain dysfunction (390, 92.9%), too much worry (388, 92.4%), accident or injury (357, 85.0%), and conflict in marriage or family (364, 86.7%). Conversely, mental illness was attributed to possession by evil spirits, God's punishment, and witchcraft by (368, 87.6%), (321, 76.4%), (259, 61.7%) of the respondents, respectively (Table 3).

**Table 3:** Knowledge of the respondents towards mental health and mental health problems at Jimma Zone, Seka woreda South-west Ethiopia, March 2020

Variables	Characteristics	Response	Frequency	Percentage
Metal illness is due to	Genetic reasons	Yes	116	27.6
	Stress/tension	Yes	377	89.8
	Accident/injury	Yes	357	85.0
	Brain functional abnormality	Yes	390	92.9
	Family events/conflict	Yes	350	83.3
	Conflict in marriage or family	Yes	364	86.7
	Worrying too much	Yes	388	92.4
	Neurotransmitter imbalances	Yes	271	64.5
	witchcraft	Yes	259	61.7
	God's punishment for past sins	Yes	321	76.4
	Evil spirit possession	Yes	368	87.6
	Personal weakness	Yes	240	57.1
	Poor nutrition	Yes	210	50.0
	Polluted atmosphere	Yes	149	35.5
Mental illness can be treated	Traditional	Yes	205	48.8
	Religious	Yes	272	64.8
	Medical	Yes	337	80.2
professional advice or counseling can be an effective treatment for people with mental illnesses		Yes	256	61.0
Medication can be an effective treatment for people with mental illnesses		Yes	358	85.2
Mental illness requires treatment from the psychiatric hospital		Yes	378	90
Mental illness can be successfully managed at home by families		Yes	209	49.8
Mental illness should be managed by witchdoctors		Yes	215	51.2
Mental illness can be cured by marriage		Yes	207	49.3

Responses only “Yes” or “No”

### Overall knowledge level of the study participants

The median overall knowledge score value was 33 (SD ± 3.55), with the minimum and maximum values of 19 and 42 out of 42 knowledge items. Calculating the proportion of the study population with a cutoff score below and above median score, the overall knowledge score showed (188, 44.8%) respondents had inadequate knowledge towards mental health problems.

### Predictors of knowledge regarding mental health problems

Variables such as sex, age, residence, birth order, family monthly income, educational status, marital status, occupational status were entered in the bivariate logistic regression analysis, and sex, residence, and educational level were found to have an association at *P*-value < 0.25. Finally, multivariate logistic regression analysis revealed, respondents who were able to read and write were 64% less likely to have adequate knowledge compared to those who were with secondary school and above educational status (AOR = 0.34, 95% CI (0.16–0.69)) (Table 4).

**Table 4:** Factors associated with mental health and mental health problems knowledge of the respondents at Jimma Zone, Seka woreda, South-west Ethiopia, March 2020

Variables	Category	Knowledge toward mental health and mental problems		COR (95% CI)	AOR (95% CI)
		Inadequate	Adequate		
		Frequency (%)	Frequency (%)		
Sex	Male	94 (49.5)	96 (50.5)	0.70 (0.47–1.04)*	
	Female	94 (40.9)	136 (59.1)	1	
Age	18–28	40 (40.4)	59 (59.6)	1.18 (0.63–2.23)	
	29–39	88 (49.7)	89 (50.3)	0.81 (0.46–1.44)	
	40–49	31 (39.2)	48 (60.8)	0.81 (0.64–2.42)	
	> 50	29 (44.6)	36 (55.4)	1	
Marital status	Unmarried	36 (48.0)	39 (52.0)	0.85 (0.51–1.40)	
	Married	152 (44.1)	193 (55.9)	1	
Residence	Urban	83 (49.7)	84 (50.3)	1.39 (0.94–2.06)*	
	Rural	105 (41.5)	148 (58.5)	1	
Birth order	First	84 (45.9)	99 (54.1)	0.78 (0.45–1.35)	
	Second	49 (48.0)	53 (52.0)	0.72 (0.39–1.31)	
	Third	25 (41.7)	35 (58.3)	0.93 (0.46–1.86)	
	4th or more	30 (40.0)	45 (60.0)	1	
Family monthly Income	< 1000	77 (40.1)	115 (59.9)	1.04 (0.38–2.86)	
	1001–2999	95 (51.1)	91 (48.9)	0.67 (0.24–1.83)	
	3000–4999	9 (36.0)	16 (64.0)	0.24 (0.35–4.40)	
	> 5000	7 (41.2)	10 (58.8)	1	
Educational Level	Unable to read and write	63 (37.5)	105 (62.5)	1.14 (0.62–2.09)	0.95 (0.51–1.78)
	Read and write	51 (63.0)	30 (37.0)	0.40 (0.20–0.80)*	0.34 (0.16–0.69)**
	Primary school (1–8)	50 (44.6)	62 (55.4)	0.61 (0.85–0.44)	0.77 (0.40–1.48)
	Secondary school (9–12) and above	24 (40.7)	35 (59.3)	1	1
Occupational status	Farmer	95 (43.4)	124 (56.6)	0.98 (0.61–1.58)	
	Merchant	30 (49.2)	31 (50.8)	0.78 (0.41–1.47)	
	Housewife	20 (50.0)	20 (50.0)	0.75 (0.36–1.57)	
	Employed	43 (43.0)	57 (57.0)	1	

1, Reference

*P-value < 0.25, **Variables significant at *P* value < 0.05

## Discussion

In the current study, nearly half of the respondents had inadequate knowledge regarding mental health and mental health problems. This finding is consistent with the studies conducted in Nigeria [37, 38, 53, 54], Tanzania [41], Southern India [28], among residents of Gimbi town, Ethiopia [9], Denmark [40], Lebanon [30], and Saudi Arabia [55]. Additionally, a report of the Ethiopian public health association expert group report indicates, limited knowledge of mental health problems is prevalent in the community [63]. The overall inadequate knowledge across all the studies could reflect that despite the high prevalence of mental health problems that affect every community across the world, less emphasis and attention has been given to design appropriate strategies to fill the knowledge gap.

This study finding has revealed a blend of biological, psychological, and cultural factors are the cause mental illness. This result was supported by the studies done in Nigeria [37–39], northern Ethiopia [13], and Denmark [40]. In contrary to this, biological factors were reported as the main causes of mental illness in the studies conducted in Tanzania [41], Eretria [38], and Saudi Arabia [42]. Additionally, religious and cultural explanations for the causes of mental illness were reported by the studies done in South Africa [43], Agro, Gimbi town, and Borana Ethiopia [44–46], Nigeria [36], and Pakistan [47]. The differences in the description of causes of mental illness across different societies could be explained by the socio-economic, literacy status, urban–rural study setting differences across the studies.

Nearly all the study participants described talking or laughing alone and showing strange or unusual behaviors were the manifestations of mental illness. This is in line with the studies done in northern Nigeria [39, 54, 64], Jeddah city Saudi Arabia [42], Agaro town, Ethiopia [52], and India [65]. This finding could reflect the similarities of mental health problem manifestations across different geographical locations of the world.

More than three-quarters of the respondents reported recovered psychiatric patients are employed productively. This finding is supported by the study done in north Ethiopia [13]. Contrary to this, the studies were done in Nigeria [37] and Tanzania [41] reported that a mentally ill patient could not be productive even after recovery. This could show that the study respondents are optimistic about the recovery of patients with mental health problems.

In this study, most of the respondents mentioned the combination of medical, religious, and traditional treatments for mental illness, and this is consistent with the studies done in Eritrea [48], Saudi Arabia [49], Nigeria [39], and Ethiopia [5]. In contrast to this, the studies were done in Tanzania [41], Saudi Arabia [42], Slovak Republic, and Russia [50], New Zealand [51], South Africa [43], Ethiopia [13, 52], and India [45] revealed medical management (medication and counseling) was identified as the first option for treatment of mental illnesses. Whereas, the studies conducted in Southern India [28] and Nigeria [38, 53, 54] have shown traditional treatments (Cultural or religious) as primary choices of treatment. Different factors such as literacy status, availability of the mental health treatment services, distance and affordability of mental health service, quality of mental health service, mental health professional availability, and religious influence may contribute to the selection of treatment options in the community.

Almost eighty-five percent of this study participants stated that mental illness is treatable; this is consistent with the studies done in Eretria, Nigeria, India [38, 45, 48], and New Zealand [51]. These findings could confirm that despite the differences in the preference of treatment options, the community is hopeful and believes mental illness better treatment outcomes. However, half of the respondents reported that mental illness could be cured by marriage in the current study. A similar finding was found in the research done in Southern India [28]. This report could reflect the community knowledge gap regarding mental health problem treatments.

In this study, the educational level of the participants was found associated with community knowledge about mental health problems. This finding is supported by the studies done in Ethiopia [9, 13] and China [66]. On the other hand, the studies were done in Saudi Arabia [55, 67], and Lebanon [30] found no association between the community knowledge about mental health problems and educational level.

## Conclusion

Knowledge of mental illness among the general public was relatively poor and significantly associated with educational status. This suggests the need for a strong emphasis on public education to improve the mental health literacy status of the general public.

## Supplementary Information

**Additional file 1**. Appendix listing survey questions.

## Data Availability

The datasets used and/or analyzed during the current study are available from the corresponding author on reasonable request.

## Abbreviations

AOR: Adjusted odds ratio
CI: Confidence interval
MAKS: Mental health knowledge schedule
SD: Standard deviation
SPSS: Statistical package for social science studies
US: United States
WHO: World Health Organization
