# Supplementary material for: Knowledge of the community regarding mental health problems: a cross-sectional study
Source: BMC Psychol. 2021 Jul 14;9:106. doi: 10.1186/s40359-021-00607-5 (PMC8278586; doi:10.1186/s40359-021-00607-5)
Supplement: Supplementary file 1 — Additional file 1. Appendix listing survey questions. [file 40359_2021_607_MOESM1_ESM.docx]

**Knowledge** **of the community regarding mental health problems. A cross-sectional study**

Yonas Tesfaye^1^, Liyew Agenagnew^1^, Susan Anand^2^, Gudina Terefe Tucho^3^, Zewdie Birhanu^4^, Gutema Ahmed,^1^ Masrie Getnet^5^_,_ Kiddus Yitbarek ^6^

^1^Department of Psychiatry, Jimma University, Jimma, Oromia, Ethiopia

^2^School of Nursing and Midwifery, Jimma University, Jimma, Oromia, Ethiopia

^3^Department of Environmental health sciences and Technology, Jimma University, Jimma, Oromia, Ethiopia

^4^Department of Health, Behavior, and Society, Jimma University, Jimma, Oromia, Ethiopia

^5^Department of Biostatistics and Epidemiology, Jimma University, Jimma, Oromia, Ethiopia

^6^Department of Health policy and management, Jimma University, Jimma, Oromia, Ethiopia

Corresponding author: Yonas Tesfaye

Po box: 378

Telephone number: +251910107507

Email: [yonastesfaye71@yahoo.com](mailto:yonastesfaye71@yahoo.com)

**Jimma University**

**Informed consent sheet**

Hello! I am ……………. from Jimma University, I am here with my colleagues to assess the knowledge of the community regarding mental health problems. I am going to give you the information and invite you to be part of this research study. Please ask me to stop as we go through the information and I will take time to explain.

**Purpose of the study**: Mental health problems are the global public health problem in the world and Ethiopia. Despite this low mental health service utilization and stigma are common in the community. The main challenge with the treatment and prevention of these disorders is the low mental health literacy status of the community particularly in developing countries like Ethiopia. We are doing this research to generate evidence for appropriate community based mental health interventions.

**Voluntary Participation:** You are randomly selected. Your participation in this research is entirely voluntary. You can stop participating in the study at any time, for any reason, if you so decide.

**Risks and discomforts**: There are no risks and discomforts associated with this interview

**Benefits**: There will be no direct benefit to you, but your cooperation will help us generate evidence for appropriate community based mental health interventions.

**Confidentiality:** The information that we collect from this research project will be kept private. We will not share any information about you with anyone outside of the research team.

Whom to contact: If you have any questions related to the study you may ask me or, kindly contact the following research team members:

1. Mr. Yonas Tesfaye: Jimma University, Jimma, Ethiopia

E-mail: yonastesfaye71@yahoo.com, Mobile No. + 251 910107507

2. Mr. Kiddus Yitbarek: Address: Jimma University, Jimma, Ethiopia

E-mail: kiddus.yitbarek@yahoo.com, Mobile No. +251 91 320 8902

Legal Rights and Signatures: I have been invited to participate in research about the assessment of "Knowledge of the community regarding mental health problems. I have read the foregoing information, or it has been read to me. I confirm my consent to participate in the study with my signature below.

N.B If the respondents unable to read and write please take the signature from a legally authorized representative

Name (participant) -------------------------Signature -------------------------Date ---------

Name (Data collector) ------------------------Signature --------------------------Date ----------

**Part I: Sociodemographic characteristics of respondents at** **Jimma Zone, Seka woreda, South-west Ethiopia, March 2020**

**Instruction: Encircle the respondent response**

| No | Characteristic | Choice | Remark |
| --- | --- | --- | --- |
| Q1 | sex | 1. Male  2. Female |  |
| Q2 | Age | _______________ |  |
| Q3 | Residence | 1.Urban  2.Rura |  |
| Q4 | Birth order | 1.first  2.second  3.third  4. 4 or more |  |
| Q5 | Ethnicity | 1. Oromo  2. Amhara  3. Yem  4. Kefa  5. Dawro  6. Other____________________ |  |
|  |  |  |  |
|  |  |  |  |
| Q6 | Religion | 1. Muslim |  |
|  |  | 2. Orthodox |  |
|  |  | 3. Protestant |  |
|  |  | 4. other_____________________ |  |
| Q7 | Family monthly Income | ___________________________ |  |
| Q8 | Educational status | 1. Unable to read and write |  |
|  |  | 2. Read and write |  |
|  |  | 3. Primary school (1-8) |  |
|  |  | 4. Secondary school (9-12) |  |
|  |  | 5. Diploma |  |
|  |  | 6. Degree and above |  |
| Q9 | Marital status | 1. Single |  |
|  |  | 2. married |  |
|  |  | 3. Divorced |  |
|  |  | 4. Widowed |  |
| Q10 | Occupational status | 1. Farmer |  |
|  |  | 2. Merchant |  |
|  |  | 3. Daily laborer |  |
|  |  | 4. Housewife |  |
|  |  | 5. Private employed |  |
|  |  | 6. Government employed |  |
|  |  | 7. Other________________ |  |

**Part II Knowledge of the community towards mental health and illness Jimma Zone, Seka woreda, South-west Ethiopia, March 2020**

| **No** | **Characteristics** |  | **Yes** | **No** | **remarks** |
| --- | --- | --- | --- | --- | --- |
| **K1** | Psychiatric disorders are a kind of medical disorders |  |  |  |  |
| **K 2** | Psychiatric disorders are contagious diseases |  |  |  |  |
| **K 3** | Recovered psychiatric patients are employed productively |  |  |  |  |
| **K 4** | Psychiatric disorders are treatable |  |  |  |  |
| **K 5** | People with severe mental health problems can fully recover |  |  |  |  |
| **K 6** | Leaving alone is the treatment for mental illness |  |  |  |  |
| **K 7** | Which are symptoms of mental illness | K 7 Irritability |  |  |  |
|  |  | K 8 Talking/laughing alone |  |  |  |
|  |  | K 9 Wandering |  |  |  |
|  |  | K 10 Excessive and unusual happiness |  |  |  |
|  |  | K 11 Strange/unusual behavior |  |  |  |
|  |  | K12 Excessive Feeling sad, tearful |  |  |  |
|  |  | K13 Aggression/violence |  |  |  |
|  |  | K 14 Hearing and seeing things which are not there |  |  |  |
|  |  | K 15 Lack of sleep |  |  |  |
|  |  | K 16 Talkativeness |  |  |  |
|  |  | K 17 Trying to kill oneself |  |  |  |
|  |  | K 18 Isolating oneself |  |  |  |
| **K.19** | Older people may develop mental disorders |  |  |  |  |
| **K.20** | Children may develop mental disorders |  |  |  |  |
| **K.21** | Women may develop mental disorders as equal to male |  |  |  |  |
| **K.22** | Lower socioeconomic class increases the risk of having psychiatric disorders |  |  |  |  |
| **K.23** | Psychiatric disorders are due to | K.23. Genetic reasons |  |  |  |
|  |  | K.24. Stress/tension |  |  |  |
|  |  | K.25. Accident/injury |  |  |  |
|  |  | K.26. Brain functional abnormality |  |  |  |
|  |  | K.27. Family events/conflict |  |  |  |
|  |  | K.28. Conflict in marriage or family |  |  |  |
|  |  | K.29. Worrying too much |  |  |  |
|  |  | K.30. Neurotransmitter imbalances |  |  |  |
|  |  | K.31. witchcraft |  |  |  |
|  |  | K.32. God's punishment for past sins |  |  |  |
|  |  | K.33. Evil spirit possession |  |  |  |
|  |  | K.34. Personal weakness |  |  |  |
|  |  | K.35. Poor nutrition |  |  |  |
|  |  | K.36. Polluted atmosphere |  |  |  |
| K.37 | Mental illness can be treated | 1.Traditional  2.Religious  3.Medical |  |  |  |
| K.38 | professional advice or counseling can be an effective treatment for people with mental illnesses |  |  |  |  |
| K.39 | Medication can be an effective treatment for people with mental illnesses |  |  |  |  |
| K.40 | Mental illness requires treatment from the psychiatric hospital |  |  |  |  |
| K.41 | Mental illness can be successfully managed at home by families |  |  |  |  |
| K.42 | Mental illness should be managed by witchdoctors |  |  |  |  |
| K.43 | Mental illness can be cured by marriage |  |  |  |  |
